# MSX1-expression during the different phases in healthy human endometrium

**DOI:** 10.1007/s00404-023-07033-5

**Published:** 2023-04-27

**Authors:** Simon Eppich, Christina Kuhn, Elisa Schmoeckel, Doris Mayr, Sven Mahner, Udo Jeschke, Julia Gallwas, Helene Hildegard Heidegger

**Affiliations:** 1 Department of Obstetrics and Gynecology, University Hospital, Ludwig Maximilians University (LMU), Marchioninistraße 15, 81377 Munich, Germany; 2 Department of Pathology, LMU Munich, Thalkirchner Str. 56, 80337 Munich, Germany; 3 Department of Obstetrics and Gynecology, University Hospital Augsburg, Stenglinstr. 2, 86156 Augsburg, Germany; 4 Department of Gynecology and Obstetrics, Georg August University Göttingen, University Medicine, Göttingen, Germany

**Keywords:** MSX-1, Progesterone receptor, Endometrium, Proliferative phase, Glandular cells

## Abstract

**Purpose:**

The human endometrium consists of different layers (basalis and functionalis) and undergoes different phases throughout the menstrual cycle. In a former paper, our research group was able to describe MSX1 as a positive prognosticator in endometrial carcinomas. The aim of this study was to examine the MSX1 expression in healthy endometrial tissue throughout the different phases to gain more insight on the mechanics of MSX-regulation in the female reproductive system.

**Materials and methods:**

In this retrospective study, we investigated a total of 17 normal endometrial tissues (six during proliferative phase and five during early and six during late secretory phase). We used immunohistochemical staining and an immunoreactive score (IRS) to evaluate MSX1 expression. We also investigated correlations with other proteins, that have already been examined in our research group using the same patient collective.

**Results:**

MSX1 is expressed in glandular cells during the proliferative phase and downregulated at early and late secretory phase (*p* = 0.011). Also, a positive correlation between MSX1 and the progesterone-receptor A (PR-A) (correlation coefficient (cc) = 0.0671; *p* = 0.024), and the progesterone receptor B (PR-B) (cc = 0.0691; *p* = 0.018) was found. A trend towards negative correlation was recognized between MSX1 and Inhibin Beta-C-expression in glandular cells (cc = − 0.583; *p*-value = 0.060).

**Conclusion:**

MSX1 is known as a member of the muscle segment homeobox gene family. MSX1 is a p53-interacting protein and overexpression of homeobox MSX1 induced apoptosis of cancer cells. Here we show that MSX1 is expressed especially in the proliferative phase of glandular epithelial tissue of the normal endometrium. The found positive correlation between MSX1 and progesterone receptors A and B confirms the results of a previous study on cancer tissue by our research group. Because MSX1 is known to be downregulated by progesterone, the found correlation of MSX1 and both PR-A and -B may represent a direct regulation of the MSX1 gene by a PR-response element. Here further investigation would be of interest.

## What does this study add to the clinical work

**Table:** 

In this study, we showed that MSX1 is highly expressed especially in the proliferative phase of glandular epithelial tissues of normal endometrium. By the found correlations of MSX1 and progesterone receptor A and B, a direct regulation of the MSX1 gene by a PR response element (PRR) can be assumed.	

## Introduction

The human endometrium consists of different layers, which are differently affected by hormonal changes [1]. There are two regions that define the subsurface endometrium: the functionalis (stratum spongiosum) and the basalis (stratum basale), with the functionalis being the tissue with the greatest degree of hormonal responsiveness [1, 2].

Due to the hormonal changes throughout the menstrual cycle the human, premenopausal endometrium undergoes different phases; there is the proliferative phase and the secretory phase [1].

The proliferative phase is mainly estrogen-dependent and starts after menses and terminates at ovulation [3, 4]. In this phase, a rapid cellular proliferation of all cell types happens and new extracellular matrix is being build up [3].

The secretory phase follows ovulation; now the progesterone-dependent differentiation of the endometrium takes place [4]. Characteristics are tortuous glands, sub-nuclear vacuoles in glandular epithelial cells, stromal oedema and maturation of spiral arterioles [4, 5].

For better understanding of this cycle and the anatomy of the female reproductive tract it is important to examine, which proteins play a potential role.

In a former analysis by our research group a possible protective effect of MSX1 in endometrial carcinomas was described [6]. Here a significantly higher expression of MSX1 especially in endometroid endometrial carcinomas was found and a MSX1-expression in more than 10% of the tumor cells was linked to better survival [6]. MSX1 is a protein, that as a transcriptional repressor plays a role during embryogenesis (especially during the formation of limb-patterns, craniofacial development, odontogenesis), but also is thought to function in tumor growth inhibition [7–10]. By examining mouse models the importance of the homeobox gene family (MSX1 and MSX2) for embryo implantation was described [11, 12]. In a study by Bolnick et al., a reduced MSX1-level was linked to infertility [13]. So, to better understand this protein’s function in the female reproductive system, the idea for this analysis was to examine the MSX1-expression in healthy endometrium throughout the different phases and to look for possible correlations with proteins, that have already been examined in the same patient collective by our research group, and by this to gain more insight on the complex biochemical mechanics of the female reproductive system.

## Materials and methods

### Patients and tissue collection

For this study, samples of healthy endometrium were used. We retrospectively analyzed a total of 19 patients. All samples were collected at the Department of Obstetrics and Gynecology of the Ludwig-Maximilians-University. Informed consent from all patients was obtained before surgery.

The material was obtained from premenopausal, non-pregnant patients undergoing gynecological surgery for benign diseases (either by dilatation and curettage or hysterectomy). No hormone therapy was applied for 3 months prior to surgery and all women had a normal and regular menstrual cycle. Endometrial samples that were pathological and/or hyperplastical were excluded from this study. Data regarding state of fertility are not available. According to anamnestical and (by using hematoxylin and eosin staining) histological data [5, 14] the samples were classified into proliferative phase (*n* = 6), early secretory (*n* = 5), and late secretory phase (*n* = 6).

The samples were all formalin-fixed and paraffin-embedded. Endometrium tissue of the different phases was used (proliferative phase, early secretory phase, late secretory phase). Table 1 shows patients’ characteristics. Surgery took place between 1 January 1990 and 31 December 2001 in the Department of Gynecology, Ludwig-Maximilians-University Munich, Germany.

**Table 1:** Patients’ characteristics (healthy endometrium), *n* = 17

Proliferative phase	6
Early secretory phase	5
Late secretory phase	6

All women were premenopausal, non-pregnant, and underwent surgery for benign diseases (either by dilatation and curettage or hysterectomy)

### Immunohistochemistry

For the immunohistochemical staining of the endometrial tissue, new samples of the original slides were taken and representative areas were selected. The paraffinized slides were put into Roticlear (a substitute-medium for xylol) for 20 min to deparaffinize the tissue. Afterwards they were put into 100% ethanol and then for another 20 min into 3% hydrogen peroxide diluted in methanol. By this the endogenous peroxidase activity was inhibited. Using a series of graded alcohols (100%, 70%, 50%) and eventually distilled water, the samples were rehydrated. By heating them up to 100 °C and cooking them for 5 min in a pressure cooker filled with an already boiling trisodium citrate buffer solution with pH = 6.0 the slides were demasked.

A blocking solution (Reagent 1 of polymer detection kit (ZytoChem Plus HRP Polymer System, Mouse Rabbit, Zytomed, Berlin, Germany)) was used to saturate the electrostatic charges in the tissue. The primary antibody was placed on the samples and incubated for 16 h at 4 °C. The antibody used was Anti-MSX1, Rabbit IgG polyclonal, concentration 0.2 mg/mL (Sigma; order number HPA073604. Sigma-Aldrich, Merck KGaA, Darmstadt, Germany), diluted at a ratio of 1:200 with PBS. The following day, the antibody-surplus was washed off and a post-block-reagent applied for 20 min. Secondary antibodies were conjugated with horseradish peroxidase (HRP) for another 30 min. Finally, the antibody was stained by applying DAB (chromogen substrate kit, Dako North America Inc., Carpinteria, CA, USA) for 5 min. The staining reaction was then stopped using distilled water and the material was counterstained with hematoxylin. Then the samples were dehydrated with an ascending series of graded alcohols (50%, 70%, 96%, and 100%) and Roticlear. As final step, the samples were covered with a mounting medium and cover glasses. This method has already been described by our research group [6].

For evaluation the IRS (immunoreactive score) was applied, which is the result of the percentage of stained cells (0 = 0%, 1 = 1–10%, 2 = 11–50%, 3 = 51–80%, 4 ≤ 81%) multiplied with the coded staining intensity [15]. In statistical analysis, all three results were looked at separately (staining intensity, percentage of stained cells, and IRS).

### Statistical analysis

The statistical analysis software SPSS (IBM SPSS Statistics, Version 25; IBM Deutschland GmbH, Ehningen, Germany) was used. For evaluation of the clinical-pathological variables, the Kruskal–Wallis test was applied, and for testing correlations we used the bivariate correlation of Spearman. For all analyses, a *p*-value < 0.05 was considered statistically significant.

## Results

**Fig. 1:**
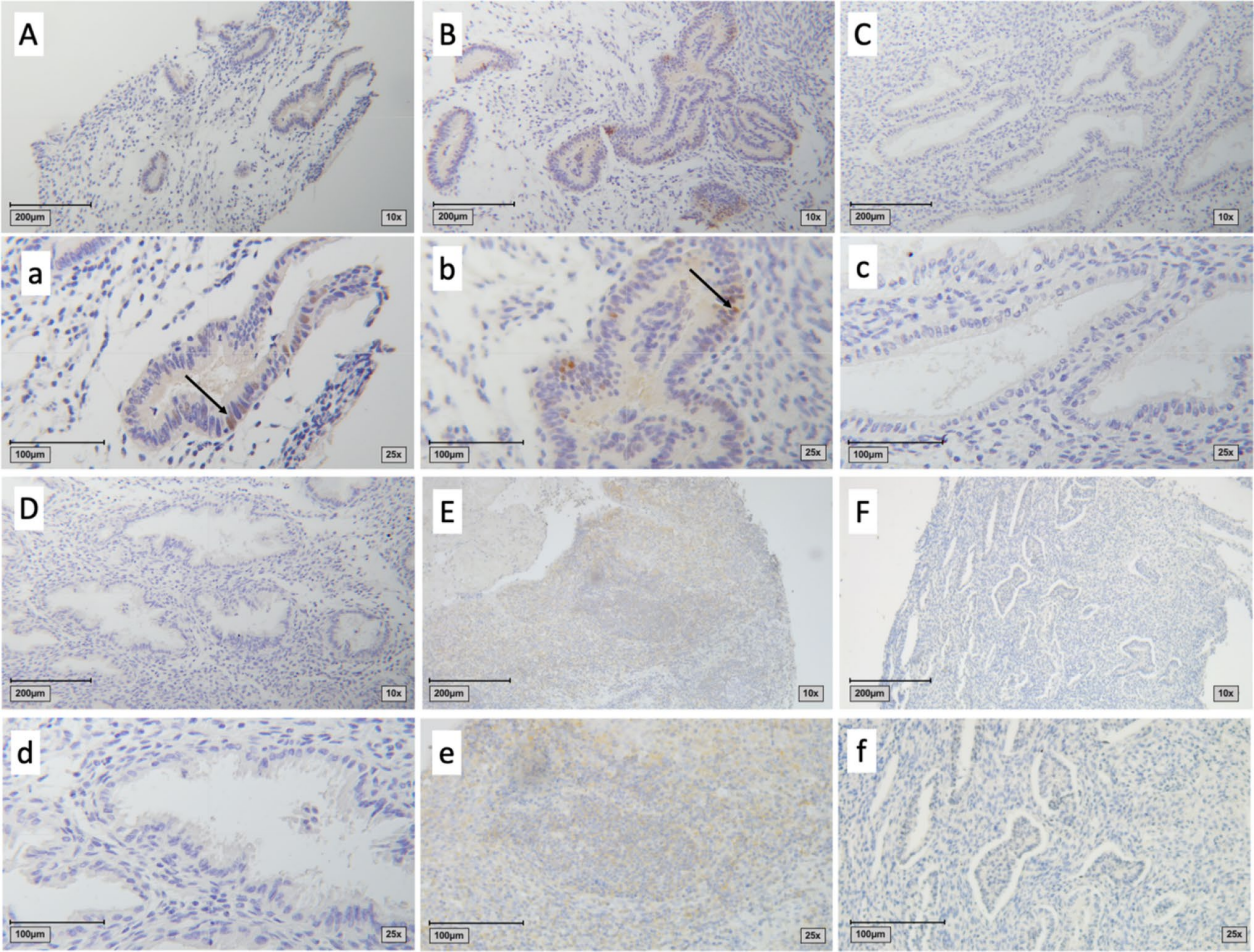
Healthy endometrium stained for MSX1. Capital letters show 10 × magnification, small letters show 25 × magnification. A/a and B/b: proliferative phase with an immunoreactive score (IRS) of 1 (arrows indicate the expressing cells; only glandular cells showed a positive staining); C/c: early secretory phase with an IRS of 0; D/d: late secretory phase with an IRS of 0; E/e: Positive control (tonsil); F/f: negative control (healthy endometrium, stained without the primary antibody)

**Fig. 2:**
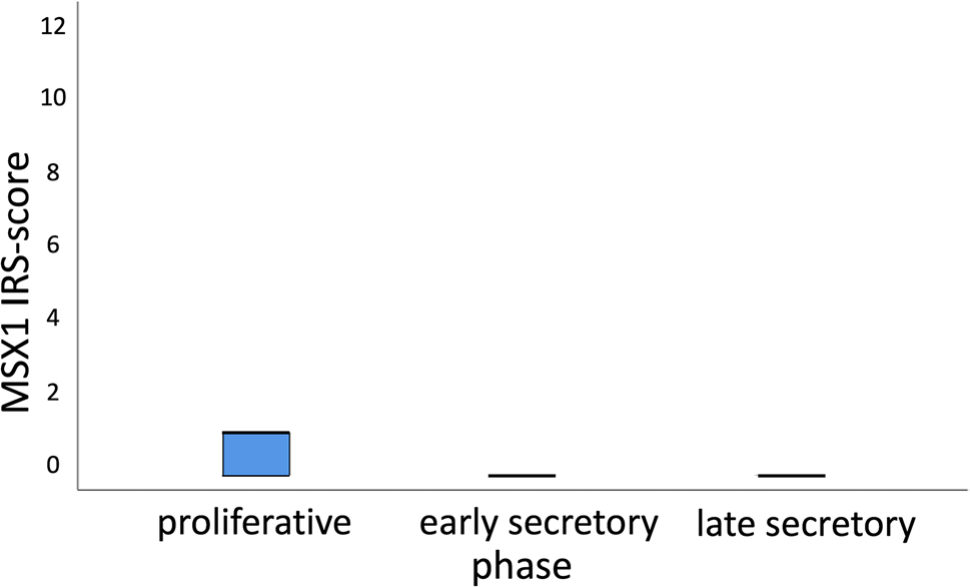
Boxplots with a median IRS of 1 for proliferative phase and with a median IRS of 0 for early and late secretory phase (*p* = 0.011)

Our results show a significant difference in MSX1 expression throughout the different phases. The specimens of endometrium during the proliferative phase show a staining in glandular cells with a mean IRS of 1 (*p* = 0.011), while the early and the late secretory phase show no staining for MSX1 at all. So even the positive stainings have a rather low IRS (range from 0 to 12) (Figs. 1 and 2).

As a number of proteins have already been investigated in this collective by our research group, we were able to check for possible correlations with MSX1 [16–19]. Interesting results were found for the progesterone-receptors A and B and Inhibin Beta-C (Table 2).

**Table 2:** Correlations between MSX1 and formerly investigated markers (cc: correlation coefficient; not all samples were suitable for evaluation in all investigated proteins due to limited assessability)

	PR-A	PR-B	Inhibin Beta-C
MSX1	IRS-Score	IRS-Score	IRS-Score
cc	0.0671	0.691	− 0.583
*p*	0.024	0.018	0.060
*n* total	11	11	11
*n* proliferative phase	6	6	3
*n* early secretory phase	4	4	4
*n* late secretory phase	1	1	4

With a *p*-value of 0.024 and a correlation-coefficient (cc) of 0.0671 a positive correlation between MSX1 and the progesterone-receptor A (PR-A) was found. Although there is no significant correlation between progesterone-receptor B (PR-B) and the menstrual cycle (*p*-value = 0.465, cc = − 0.110) or Inhibin Beta-C and the menstrual cycle (*p*-value = 0.298, cc = 0.165), the following correlations with MSX1 were found: a significant positive correlation was found between MSX1 expression in glandular cells and PR-B-expression in stroma-cells (*p*-value = 0.018; correlation-coefficient cc = 0.691). Also a trend towards negative correlation was recognized between MSX1 and Inhibin Beta-C expression in glandular cells (*p*-value = 0.060; cc = − 0.583).

Therefore, we were able to confirm the results of our former paper on cancer tissue, where a significant positive correlation between MSX1 and the progesterone-receptors A and B was also found. [6]

**Fig. 3:**
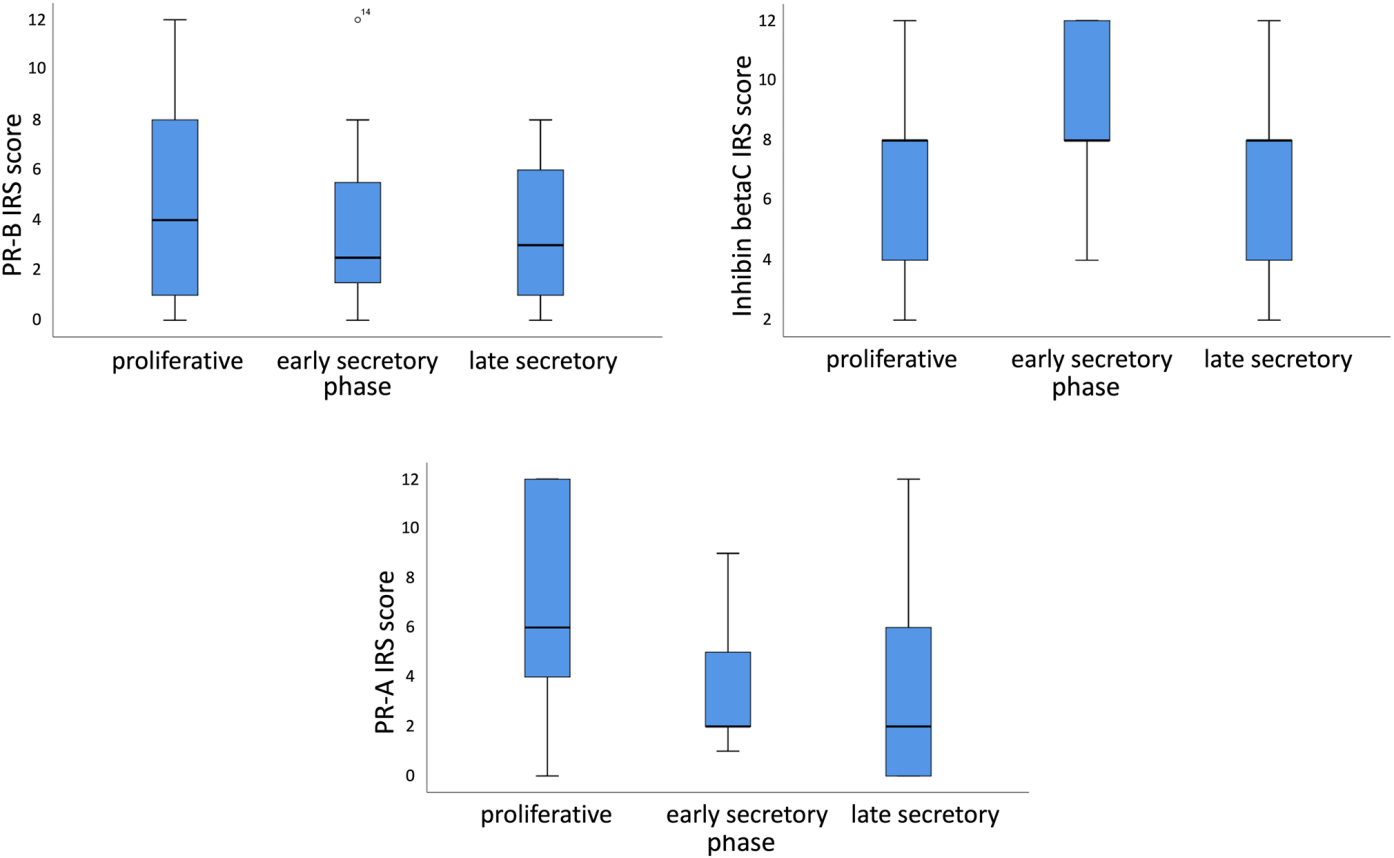
Top left: boxplots showing a median IRS of 4 for proliferative phase, 2.2 for early secretory and 3 for late secretory phase for PR-B expression in stromal cells (*n* = 42; *p* = 0.752). Top right: boxplots showing a median IRS of 8 for all phases for Inhibin Beta-C in glandular cells (*n* = 42; *p* = 0.157). Below: Boxplots showing a median IRS of 6 for proliferative phase and 2 for early and late secretory phase for PR-A (no further differentiation) (*n* = 47; *p* = 0.015)

Because of the found significant correlations between MSX1 and other already published markers of our research panel [18, 20–23], we analyzed the expression of PR-A, PR-B and Inhibin Beta-C in the menstrual cycle. The results of this analyses are presented in Fig. 3.

## Discussion

In our study, we found that MSX1 is expressed in glandular cells at a mean IRS of 1 (out of 12) during the proliferative phase and significantly downregulated at early and late secretory phase (*p* = 0.011). In addition, a positive correlation between MSX1 and the progesterone-receptor A (PR-A) (correlation coefficient (cc) = 0.0671; *p* = 0.024) and the progesterone receptor B (PR-B) (cc = 0.0691; *p* = 0.018) was found.

Blonick et al. found in their study a MSX1 accumulation during the secretory phase in comparison to the late proliferative phase. Here the focus of investigation was on embryo implantation and therefore, the period of receptivity for implantation. In our study, we looked at the different phases of the menstrual cycle; differentiation in relation to implantation or the preimplantational phase did not take place and therefore the proliferative phase was not further differentiated [13].

Progesterone regulation of the endometrial MSX1 gene was first described in 2008 in the ovine uterus [24]. In that study, Satterfield et al. [24] described that MSX1 mRNA was decreased by P4 treatment. In addition, another animal based study found that progesterone inhibits uterine gland development in the neonatal mouse uterus and downregulated MSX1 [25]. A very recent study on the role of MSX1 in reproduction was carried out on mice embryonic diapause [26]. Embryonic diapause in mice is a reproductive strategy in which embryo development and growth are temporarily halted in utero to ensure neonatal and maternal survival in adverse external conditions [26]. In that study, the authors have shown that dormant blastocysts are recovered from these mice on day 8 of pregnancy with persistent expression of uterine MSX1, a gene critical to maintaining the uterine quiescent state [26]. Interestingly, progesterone and anti-estrogen can prolong uterine quiescence [26].

In cancer biology, on the other side MSX1 was identified as a key candidate for progestin resistance in endometrial cancer [27]. MSX1 showed significant tissue specificity and better prognostic value and its knockdown enhanced progesterone efficacy [27]. Our own investigation on MSX1 in endometrial cancer showed that a better survival was identified for patients with an MSX1 expression in more than 10% of the tumor cells [6]. We further have given the hypothesis that MSX1 could be a potential marker for a potential uterus-preserving therapy of endometrial carcinomas [6]. In agreement to our results, high methylation and low expression of MSX1 were significantly associated with reduced endometrial cancer survival rates [28]. A similar role of MSX1 was identified for other gynecologic cancer subtypes [29]. Overexpression of MSX1 inhibited cell proliferation [29]. Furthermore, MSX1 triggers G0/G1 arrest and apoptosis [30]. In breast cancer, MSX1 inhibits breast cancer cell growth and metastasis and is often silenced by promoter methylation [31].

In addition to our progesterone regulation of MSX1, a trend towards negative correlation was recognized between MSX1 and Inhibin Beta-C-expression in glandular cells (cc = − 0.583; *p*-value = 0.060). Although no direct involvement of inhibin on MSX1 is known, MSX1 represses the αGSU and GnRH receptor genes during gonadotropic development [32]. Suppression of the mouse GnRHR promoter by MSX1 is mediated by a consensus binding motif in the downstream activin regulatory element (DARE) [32]. Activin and inhibin share the same beta subunit [33, 34].

Taken together, MSX1 is known as a member of the muscle segment homeobox gene family. MSX1 is a p53 interacting protein and overexpression of MSX1 homeobox induces apoptosis of cancer cells. Here, we show that MSX1 is expressed especially in the proliferative phase of glandular epithelial tissues of normal endometrium. We were also able to confirm the positive correlation between MSX1 and progesterone receptors A and B found in a previous study on cancer tissue by our research group. Since MSX1 is known to be downregulated by progesterone, the found correlation of MSX1 and PR-A and -B may indicate a possible direct regulation of the MSX1 gene by a PR response element (PRR). Here, further investigation would be of interest, especially as the correlation with PR-B was in relation to the stromal expression, while MSX1-expression was in glandular cells only.

### Strengths and limitations of this study

Due to the number of various proteins that have already been investigated in this collective by our research group, we have a wide variety of possible correlations that can be examined. The rather small collective-size, however, surely is the main limitation of this study. Nevertheless, this may provide the impetus for further investigations.
